# Unified Behavioral Scoring for Preclinical Models

**DOI:** 10.3389/fnins.2020.00313

**Published:** 2020-04-07

**Authors:** David J. Harrison, Hugo D. J. Creeth, Hannah R. Tyson, Raquel Boque-Sastre, Anthony R. Isles, Rupert Palme, Chadi Touma, Rosalind M. John

**Affiliations:** ^1^Preg Lab, School of Biosciences, Cardiff University, Cardiff, United Kingdom; ^2^Behavioural Genetics Group, Neuroscience and Mental Health Research Institute, School of Medicine, Cardiff University, Cardiff, United Kingdom; ^3^Unit of Physiology, Pathophysiology and Experimental Endocrinology, Department of Biomedical Sciences, University of Veterinary Medicine, Vienna, Austria; ^4^Department of Behavioural Biology, University of Osnabrück, Osnabrück, Germany

**Keywords:** behavior, methods, anxiety, sociability, modeling, reproducibility, testing

## Abstract

Preclinical mental health research relies upon animal models, and whilst many encouraging advances are being made, reproducibility and translational relevance may be limited by sub-optimal testing or model choices. Animal behaviors are complex and test batteries should be designed to include their multifaceted nature. However, multiple behavioral testing is often avoided due to cost, availability or statistical rigor. Additionally, despite the disparity in the incidence of mental health problems between the sexes, a move toward reducing animal numbers could be a deterrent to including both male and female animals. The current study introduces a unified scoring system for specific behavioral traits with the aim of maximizing the use of all data generated whilst reducing the incidence of statistical errors. Female and male mice from two common background strains were tested on behavior batteries designed to probe multiple aspects of anxiety-related and social behavioral traits. Results for every outcome measure were normalized to generate scores for each test and combined to give each mouse a single unified score for each behavioral trait. The unified behavioral scores revealed clear differences in the anxiety and stress-related, and sociability traits of mice. Principle component analysis of data demonstrated significant clustering of animals into their experimental groups. In contrast, individual tests returned an ambiguous mixture of non-significant trends and significant effects for various outcome measures. Utilizing a range of behavioral measures and combining all outcome measure data to produce unified scores provides a useful tool for detecting subtle behavioral traits in preclinical models.

## Introduction

Mental health disorders, such as anxiety and depression, constitute one of the main causes of disease burden worldwide (Vos et al., 2015), and their prevalence in the United Kingdom is growing (Martín-Merino et al., 2009; Fineberg et al., 2013).

Behavioral disruption related to environmental or genetic changes are commonly evaluated through the use of animal models (Steimer, 2011; Rossignol and Frye, 2012), although often the methods and tests used are suboptimal, leading to mixed results and findings that may not translate well (Perel et al., 2007; Open Science Collaboration., 2015). Studies may use a single behavioral probe to represent complex behavioral traits, whereas behavioral outcomes are a culmination of a multifaceted system, are frequently subtle, and have aspects which can present in different ways (Ferreri et al., 2011). This could lead to subtle behavioral changes being missed, or anomalous data being given undue prominence. A targeted battery of behavioral tests can give insight to a greater number of behavioral traits and therefore give a more accurate representation of the specific behaviors being studied. However, a consequence of multiple testing is an increase in the probability of generating type I statistical errors and for this reason many studies restrict the number of tests performed (Shaffer, 1995).

The current study presents a novel method of utilizing a broader battery of tests to produce a simple score to represent each behavioral trait under investigation. Such unified rating scales are well established in the clinical assessment of symptom progression for diseases such as Parkinson’s or Huntington’s (Martínez−Martín et al., 1994; Huntington Study Group., 1996), whereby patients undergo a battery of assessment measures that are scored and classified into functional groups, such as cognition and motor function. These scores are used as a scale against which different aspects of disease progression or intervention can be monitored. Changes in mental health status may be incremental and not necessarily have statistical significance for any individual test measure, but together may lead to a general improvement in one or more behavioral traits or in general wellbeing. Here, a unified scoring model is applied to detect differences in anxiety- and sociability-related behaviors between two common mouse strains, with the aim of establishing a simplified, statistically sound method of comparing complex behaviors in preclinical models.

Despite changes in behavior being the primary focus of many studies of mood disorders, the background strain used is often determined by factors other than behavioral phenotype. Reasons for strain selection are not often discussed in published behavioral methods but understandably may be attributed to availability, ease of breeding, handling or genetic manipulation, desirable *in vitro* qualities, or simply being a vestige of previous studies probing non-behavioral phenotypes (Crawley et al., 1997). Many studies have shown significant strain differences in cognitive, social, emotional- and psychological-like behavioral characteristics (Liu and Gershenfeld, 2001; Võikar et al., 2001; Abramov et al., 2008; Johnson et al., 2010; Tuttle et al., 2017), highlighting the importance of using appropriate strains for the phenotypes behavioral studies are seeking to model. The prospects of detecting subtle changes in behavior, for example epigenetic manipulations or environmental influences on psychological-like traits, are decreased when experimental groups are compared to controls with a lower phenotypic baseline. For instance, where an increase of anxiety-like behaviors is hypothesized, manifestation is more likely to be detected in a non-anxious model, and/or populations which exhibit minimal variation in the targeted phenotype. Conversely, if a strain is inherently stressed, ceiling effects could mask detection of subtle increases in stress levels. Implementing the proposed scoring method can identify traits which support the selection of one strain over another for studies probing these behaviors.

In addition to strain differences, the present study utilizes the unified scores to compare behavioral traits of female and male mice. It is common for preclinical studies to use only male animals, despite many clinically diagnosed behavioral and emotional disturbances manifesting disparately between the sexes (Blanchard et al., 1995). Prevalence and scale of anxiety disorders and major depressive disorders, for example, is greater in females compared to males, whereas signs of autism spectrum disorder and attention deficit hyperactivity disorder are more prevalent in males (Romans et al., 2007; McLean et al., 2011; Willcutt, 2012; Loomes et al., 2017). This sexual dimorphism of behavioral disorder symptomatology underlines the necessity to include both sexes in any preclinical studies, indeed the National Institutes of Health policies stipulate that all preclinical work should include both sexes or a valid explanation as to why not.

The current study introduces a novel data-inclusive analysis of a comprehensive anxiety-related and social behavioral battery designed to produce a robust method of measuring subtle behavioral traits of two widely used background strains in behavioral research, the C57BL6/J and 129S2/SvHsd mouse strains. These particular behavioral traits and mice were selected to assess the validity of the unified scoring method presented here as there is a rich history of comparative studies against which the results can be validated (Hughes, 1989; Crawley et al., 1997; Montkowski et al., 1997; Belzung and Griebel, 2001; Liu and Gershenfeld, 2001; Võikar et al., 2001; Touma et al., 2004; Sankoorikal et al., 2006; Abramov et al., 2008; Yang et al., 2011; Lorenz et al., 2013). The objective is to provide a simple output for a complex system, which minimizes the risk of type I and type II statistical errors and increases reproducibility in preclinical behavioral neuroscience. In addition, this strain and sex comparison will provide a guide to strain and/or behavioral test selection aimed to help customize future experimental design to maximize useful output and consequently reduce animal use.

## Materials and Methods

### Ethics Statement

All experiments were conducted in accordance with the ARRIVE guidelines and the United Kingdom Animals (Scientific Procedures) Act of 1986 and local ethical review under project license 30/3134.

### Animals

C57BL6/J female (*n* = 20) and male (*n* = 20) mice (hereafter referred to as BL6(F) and BL6(M) respectively), and 129S2/SvHsd female (*n* = 15) and male (*n* = 18) mice [hereafter referred to as 129(F) and 129(M) respectively], were used in this experiment. All mice were run through the full test battery described below, except for corticosterone metabolite analysis, where a random selection of animals from each group were tested (*n* = 12). Additionally, CD1 female (*n* = 4) and male (*n* = 1) mice were used as ‘host’ mice for social interaction tests. Test subjects were obtained from breeding setups of two females to one male per cage. When visible signs of pregnancy were identified [∼embryonic day 16 (E16.5)], the female mice were removed and housed in pairs until littering. All mice were housed in the same room (temp = 21 ± 2°C, humidity = 60 ± 5%, light:dark cycle 12:12 h). On weaning (P28), subject mice were housed in groups of 5 (plus one box of 3), in home cages 45 × 12 × 12cm containing sawdust, two cardboard tubes, a wooden chew stick and two squares of bedding material. *Ad libitum* standard chow food and tap water were available throughout the study. All tests were carried out during the light period (7am – 7pm) and performed by both male and female researchers. Mice were handled by either tunnel or open hand technique to reduce potential anxiety-related effects of researcher influence on behavior (Hurst and West, 2010; Clarkson et al., 2018).

### Behavioral Testing

Automated tracking software (EthoVision XT 13, Noldus, Tracksys Ltd., United Kingdom, RRID:SCR_000441 and RRID:SCR_004074) was used to blindly analyze videos of the elevated zero maze, light/dark box test, 3-chamber test and social odor discrimination.

Three tests of anxiety and stress-related behavior, and four tests of sociability were performed. Test order, each outcome measure and the behavioral trait which they probe is listed in Table 1.

**TABLE 1:** List of tests (presented in order of testing) and measures incorporated into the unified behavioral scores, which behavioral trait they are probing and the direction of influence.

Test	Measure	Score contribution	Influence
Direct social interaction (Age 4 weeks)	- Exploration time	Sociability	Excluded
	- Time following host		+ve
	- Time being followed		+ve
	- Time sniffing host		+ve
	- Time self-grooming		−ve
	- Time attacking host		+ve
	- Time being attacked		+ve
	- Time immobile		−ve
Social odor discrimination (Age 8 weeks)	- Increase in frequency of visits to novel social odor	Sociability	+ve
	- Increase in time spent sniffing novel social odor		+ve
Social propinquity (Age 9 – 12 weeks)	- Latency to first share	Sociability	−ve
	- Time vacant		−ve
	- Duration of double occupancy		+ve
Elevated zero maze (Age 12 – 13 weeks)	- Crosses into open	Anxiety	−ve
	- Time in open		−ve
	- Latency to enter open		+ve
Light/dark box (Age 18 – 20 weeks)	- Crosses into light	Anxiety	−ve
	- Time in light		−ve
	- Latency to enter light		+ve
3-chamber test (Age 20 – 22 weeks)	- Crosses into empty	Sociability	−ve
	- Crosses into occupied		+ve
	- Time in empty		−ve
	- Time in occupied		+ve
	- Latency to enter occupied		−ve
	- Mean time in occupied		+ve
Fecal corticosterone metabolites (FCMs) (Age 22 weeks)	- Change in FCM levels under stressed conditions	Anxiety/Stress	+ve

#### Anxiety and Stress-Related Tests

##### Elevated zero maze

An elevated zero maze consisting of a ring-shaped laminated wooden platform (diameter 60 cm, width 5 cm) elevated to a height of 50 cm from ground level was used to probe anxiety-type behavior. Tall walls (22 cm high) either side of the platform enclosed two opposite quarters of the zero (sheltered), with the remaining two quarters left with no side edge (anxiogenic). The maze was placed in the center of a large dimly lit (<30 lux) testing room and a high-definition video recorder mounted directly above. At the start of each trial the subject mouse was placed within a sheltered section of the maze and its movements recorded for five minutes after which the animal was removed, and the maze cleaned with 70% ethanol solution and dried before the next subject was introduced. Time spent in the anxiogenic sections, latency to enter an anxiogenic section and number of crosses between sections were recorded. In addition, the proportion of individuals in each group to remain solely in the sheltered section was calculated.

##### Light/dark box

**FIGURE 1:**
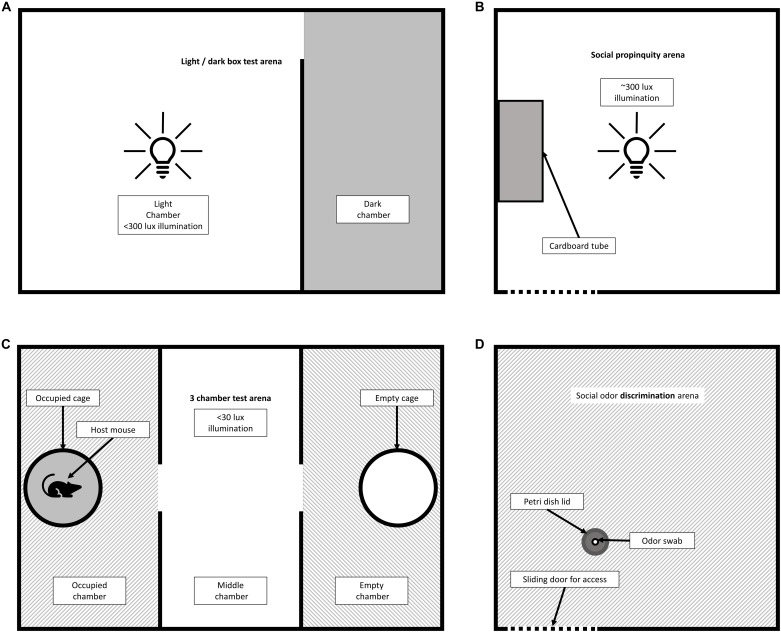
Plan view of behavioral chamber set ups. **(A)** Light/dark box test. A lamp was used to illuminate the light chamber of the box (>300 lux) whilst the smaller dark chamber remained in shadow. At the beginning of the trial, subjects were placed in the far end of the dark chamber. The subject was considered to be within the light chamber if the head (or more) was within the white chamber. **(B)** Social propinquity test. A cardboard tube was affixed to the middle of one chamber wall. **(C)** 3-chamber test. A host mouse was placed with a mesh container in one of the end chambers. At the start of each trial the subject was introduced into the middle chamber. **(D)** Social odor discrimination test. Odor swabs are placed into and removed from the petri dish via the sliding access door to the front of the chamber. Time spent sniffing and number of approaches were measured when the subject’s nose was within the perimeter of the petri dish.

Adapted from Bourin and Hascoët (2003), the light/dark box test probes anxiety-related behaviors and was conducted using a box comprising of two adjoining acrylic chambers, one black (15 × 30 × 30 cm) and one white (30 × 30 × 30 cm) separated by a dividing wall with an open doorway (5 × 5 cm), Figure 1A. The box was placed in the center of a large testing room with a high-definition video recorder mounted directly above, and a bright (∼300 lux) lamp shining directly into the white chamber (anxiogenic) ensuring that no shadows were cast within the chamber, and that the black chamber was completely in shade (sheltered). At the start of each trial a mouse was placed at the far end of the sheltered chamber and recorded for five minutes, before being removed and the chambers cleaned. The total time spent in the anxiogenic chamber, the latency to enter the anxiogenic chamber and the number of entries made were recorded.

##### Analysis of fecal corticosterone metabolites (FCMs)

Fecal samples were collected to non-invasively measure levels of the stress hormone corticosterone via its metabolites, under baseline and stress-inducing conditions. Baseline samples were collected after mice were left unhandled for 48 h in their home cages, and stress samples were collected after exposure to predator odor in home cages overnight. Predator odor was prepared by sealing bedding sawdust (20 l) in a bin bag for 48 h with 10 squares of blotting paper (5 cm^2^) each infused with 50 μl of fox odor solution (50 μl TMT, 2,4,5-Trimethylthiazole 98%, 219185-5G, Sigma-Aldrich, United Kingdom), 420 μl water and 30 μl Tween-20 (P1379-100ML, Sigma-Aldrich, United Kingdom). For both baseline and stress-conditions mice were placed into empty individual cages from 9 am – 12 pm on the morning of sample collection, with fecal pellets being collected every 90 min and stored at −20°C until processing. FCMs were subsequently extracted and quantified with a 5α-pregnane-3β,11β,21-triol-20-one enzyme immunoassay, previously described in detail and fully validated for mice (Touma et al., 2003, 2004). NB, due to the 8–12 h delay between a stress event and subsequent excretion of FCMs, separation of mice into individual unfamiliar cages would not affect the FCM levels of the baseline samples (Touma et al., 2003, 2004).

#### Social-Related Tests

##### Direct social interaction

At 4 weeks of age, i.e., before sexual maturity, direct social interaction behavior was observed within four empty phenotyper boxes (30 × 30 × 40 cm, Noldus, United Kingdom, RRID:SCR_004074). The sides of the phenotypers were obscured with dark-colored paper to reduce brightness and to prevent mice from seeing into other boxes. An adult female CD1 ‘host’ mouse was introduced the box approximately one minute prior to the test mice, and their interactions recorded for three minutes using a camera mounted in the lid of the phenotyper and EthoVision XT 13 software (Noldus, Tracksys Ltd., United Kingdom, RRID:SCR_004074).

Videos were subsequently scored using Behavioral Observation Research Interactive Software (BORIS, Friard and Gamba, 2016), and the duration for which each behavior was presented (i.e., following host, sniffing host, being followed by host, attacked by host, attacking host, self-grooming, immobile, exploration of cage) was calculated.

##### Three-chamber test

In an adaptation of the design and protocols described by Yang et al. (2011), the three-chamber test was used to assess sociability, Figure 1B. A black acrylic box divided into three chambers (of dimensions 15 × 30 × 30 cm) connected by openings (5 × 5 cm) in the middle of the longest side, was placed in the center of a large, dimly lit (<30 lux) testing room beneath an overhead high-definition video camera. A 5 mm gap between chambers allowed space for plastic sliding doors of dimensions 7 × 40 cm to slot between and block the openings. White paper was used to line the bottom of each chamber to improve the contrast between the mouse and background for the purposes of automated video analysis later. Identical up-turned wire mesh containers [10 × 9 cm (h x Ø), LAAT, China], were placed in the center of the two end chambers. A ‘host’ mouse of matching sex to the test subject was placed under one container (host side balanced between groups), which was labeled for identification during video analysis. The test subject was placed in the center of the middle chamber, with the doors closed. At the start of the trial the doors were simultaneously removed allowing the test mouse to fully explore the chambers. After five minutes the mice were removed, and the chambers cleaned with 70% ethanol with fresh paper laid down. The total length of time spent in each chamber, number of entries and latency to enter were recorded.

##### Social propinquity

Three clear plastic cages measuring 30 × 30 × 40 cm were placed on top of a clear plastic shelf and brightly illuminated from below (∼300 lux) to create an aversive arena space, in a setup adapted from Tuttle et al. (2017), Figure 1C. A cardboard tube [12 × 5 cm (L x Ø)] was secured to one edge of the arena to provide a sheltered space and opaque barriers were placed between cages to prevent mice from different cages seeing each other. Unrelated and non-cage mate mice were paired based on similar sex, strain, and weight and placed into the arena together for one hour and recorded using an overhead camera mounted on the lid of the cages. Between each trial the arena was cleaned with 70% ethanol and a fresh cardboard tube put in place.

The videos were subsequently analyzed and the latency for the first mouse to enter the tube and latency to the first time both mice cohabited the tube were recorded. The video was paused at five-minute intervals and the number of mice occupying the tube was recorded as either 0 (vacant), 1 (single occupancy) or 2 (double occupancy). From this, an approximation of the percentage of total trial time for each condition and the proportion of pairs cohabiting at each time-point were calculated.

##### Social odor discrimination

Four phenotyper cages, as described above, were used to assess social odor discrimination. White paper was used to obscure the lower half of the clear plastic cage sides and the room lights dimmed (<30 lux). The lid of a 2.5 cm diameter plastic petri dish (Fisher Scientific, United Kingdom, RRID:SCR_008452) was fixed toward the center of each arena, within reach of a sliding door at the front of the chamber, through which odors would be introduced, and the floor of the cage covered with fresh sawdust, Figure 1D. A set of cotton buds cut down to 1 cm in length were swabbed around a home cage of mice of the opposite sex and different strain (minimum of 3 days since last clean, and four mice per cage) and sealed in a falcon tube. A second home cage was swabbed with an additional set of cotton buds. Subject mice were habituated to the arena for approximately 5 min before being presented with the first odor.

The test comprised of three trials during which three odors (water, social odor cage 1, social odor cage 2) were presented three times (different swabs, same odor) for 2 min, with each swab presented individually. Each swab was placed through a 2 mm hole drilled through the center of the lower portion of the 2.5 cm petri dish with the odor upwards and fixed in place with blu-tac. Video recording was started, and the odors quickly placed into the lid in the center of the arena. At the end of the 2 min the recording was stopped, and the odor removed. After an interval of 1 min the next swab was inserted. Cages were cleaned with 70% ethanol and lined with fresh sawdust between animals.

EthoVision XT 13 software (Noldus, Tracksys Ltd., United Kingdom RRID:SCR_004074) was used to detect when the subject’s nose was within the perimeter of the odor petri dish and to calculate the latency to first approach, total time spent sniffing, and total number of visits to each odor.

### Data Transformation, Calculation of Unified Scores and Statistics

Outcome measures for each test were normalized to obtain a ‘measure score’ between 0 (low anxiety/sociability) and 1 (highly anxious/sociable) for each individual, using the following formula:

$$X\,\left(i\right)=\frac{M\,\left(i\right)}{M\,\left(m\right)}$$

where *X*(*i*) = normalized individual measure score, *M*(*i*) = actual individual measure datum [e.g., time spent in light (s)], and *M(m)* = maximum measure datum in study cohort (all mice). Negative measure data values were assigned a score of 0, and time-out scores (e.g., failure to enter light) received a latency measure score of 1.

Principal component analyses (PCAs) were performed on measure scores for each multiple-output test using the protocols outlined by Zaiontz (2019). The components contributing to the greatest variance within each test (i.e., PC1 and PC2, the factors accounting for the greatest and second greatest variance respectively) were plotted and the principle component which best explained how each measure represented the behavioral traits being probed was used to validate the allocation of measures as either a positive or negative factor, such that a positive factor *increases* as anxiety increases (e.g., latency to enter light), whilst a negative factor *decreases* as anxiety increases (e.g., time spent in light).

For measures determined to be negative factors, the measure score was subsequently inverted using the formula below, thus ensuring a greater score in any measure related to an increase in the specified behavior.

$$X\,\left(i\right)=1-\frac{M\,\left(i\right)}{M\,\left(m\right)}$$

Individual ‘test scores,’ ***T(i)***, were calculated from the mean ***X(i)*** of all outcome measures associated with that test. Unified anxiety and sociability scores were subsequently calculated for individual mice as the mean of all ***T(i)***. This enabled all outcome measures of each test to contribute to the score, with each test having an equal influence on the final result. Datasets of raw, transformed and unified data are available (Figshare,)

Finally, PCA was used to assess the clustering of individuals based on the all behavioral tests conducted.

### Statistics

Data were analyzed using GenStat 19th edition (VSN International, United Kingdom) using two-way ANOVA and Newman–Keuls *post hoc* test where appropriate.

## Results

Male and female C57BL6/J and 129S2/SvHsd mice were tested on a battery of anxiety- and sociability-related tasks and scored on cohort-normalized scales between 0 and 1, whereby a score of 0 represents the lowest measured anxiety/sociability signs and a score of 1 represents the highest.

**FIGURE 2:**
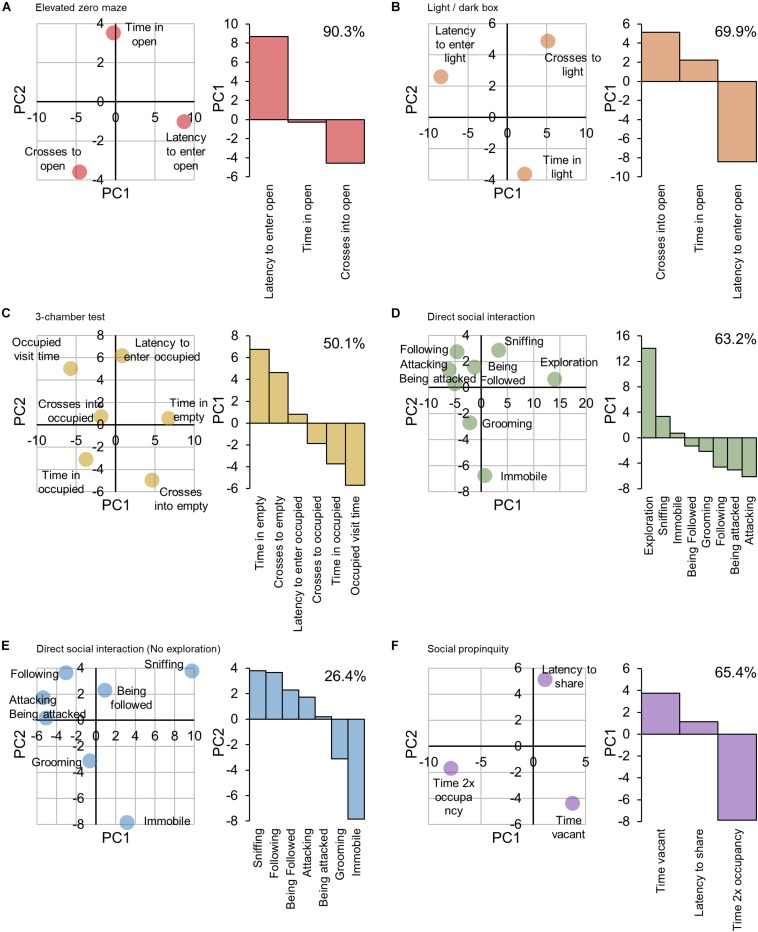
Principle component analyses of outcome measures for each multi-factor behavioral test. The principle components explaining the greatest and second greatest variance within the data (PC1 and PC2 respectively) were used to validate the allocation of the outcome measures of each test as either contributing positively (i.e., greater measure score = higher anxiety or sociability score) or negatively (i.e., greater measure score = lower anxiety or sociability score) toward the unified score. **(A)** Elevated zero maze. PC1 accounting for 90.3% of variance, reflected the directional influence of anxiety-related factors, with the ‘latency to enter the open section’ outcome measure (PC1 +ve) opposing the ‘time spent in the open section’ and ‘number of crosses into the open section’ measures (PC1 –ve), and was thereby considered to be representative of the ‘anxiety-related’ score. Therefore, the ‘latency’ measure was allocated a positive contribution to the anxiety score, and both the ‘time spent’ and ‘number of crosses’ were allocated an inverse contribution. **(B)** Light/dark box. PC1, accounting for 69.9% of variance, reflected the directional influence of anxiety-related factors, with ‘latency to enter the light chamber’ of the box (PC1 –ve) opposing the ‘time spent in’ and ‘number of crosses into’ the light chamber’ (PC1 +ve). **(C)** 3-chamber test. PC1, accounting for 50.1% of variance, reflected the directional influence of sociability-related factors, with the ‘time spent in the empty chamber,’ ‘number of crosses into the empty chamber’ and ‘latency to enter the occupied chamber’ (PC1 +ve) opposing ‘the number of crosses into the occupied chamber,’ ‘total time spent in the occupied chamber’ and ‘mean time spent per visit in the occupied chamber’ (PC1 –ve). **(D)** Direct social interaction. PC1, accounting for 63.2% of variance, did not reflect the directional influence of sociability-related factors, indicating that this represents an extraneous factor to sociability. **(E)** Direct social interaction minus ‘exploration.’ PC1, accounting for 42.5% of variance, did not reflect the directional influence of sociability-related factors, indicating that this represents an extraneous factor to sociability. However PC2, accounting for 26.4% of variance, did reflect the directional influence of sociability-related factors, with the time spent interacting with the host (‘sniffing,’ ‘following,’ ‘being followed,’ ‘attacking’ and ‘being attacked’) (PC2 +ve) opposing the time spent self-grooming or immobile (PC2 –ve). **(F)** Social propinquity. PC1, accounting for 65.4% of variance, reflected the directional influence of sociability-related factors, with the ‘duration the tube was vacant’ and the ‘latency to share the tube’ (PC1 +ve) opposing the time spent sharing the tube (PC1 –ve).

Principal component analyses of outcome measures for each multi-outcome test were used to validate their assignment as contributing either positively (higher score, greater anxiety/sociability) or negatively (higher score, low anxiety/sociability) to the behavioral trait scores being probed, Figure 2. For the elevated zero maze, PC1 described the ‘latency’ measure as contributing contrariwise to the variance compared to the ‘number of crosses’ and time spent in the open’ measures (+ve, −ve, and −ve respectively, Figure 2A), i.e., a large score in the latency measure would indicate a *higher* anxiety score, whereas a large score in the other measures would indicate a *lower* anxiety score. PC1 was also able to demonstrate the opposing contributions of the ‘latency’ measure and ‘number of crosses’ and ‘time to cross’ measures of the light/dark box test to the anxiety score, Figure 2B. The sociability outcome measures of the 3-chamber test were grouped by PC1 into ‘time spent in the empty chamber,’ ‘crosses into the empty chamber’ and ‘latency to enter the occupied chamber’ versus ‘crosses to,’ ‘time spent’ and ‘time per visit’ in the occupied chamber, Figure 2C. For the direct social interaction test, PC1 did not reflect the directional influence of sociability-related factors, indicating that this represents an extraneous factor to sociability, Figure 2D, with an environmental exploration component providing a more feasible explanation. Since it did not appear to be relevant to measures of sociability, the ‘exploration’ measure was removed and the PCA repeated, Figure 2E. PC2 reflected the directional influence of sociability-related factors, with the time spent interacting with the host (‘sniffing,’ ‘following,’ ‘being followed,’ ‘attacking’ and ‘being attacked’) (socially interactive factors) opposing the time spent self-grooming or immobile (socially anxious factors). For the social propinquity test, PC1 was descriptive of the directional influence of sociability-related factors, with the duration the tube was vacant and the latency to share the tube (socially anxious factors) opposing the time spent sharing the tube (socially interactive factor). The allocation of outcome measures to positive or negative score contributions is summarized in the ‘Influence’ column of Table 1.

**FIGURE 3:**
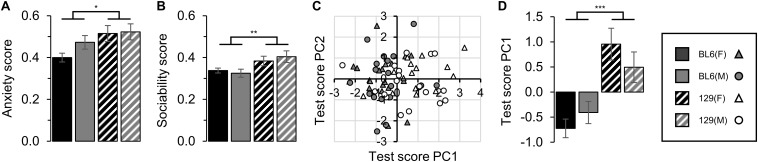
Unified scoring. **(A)** Unified anxiety score. The 129 anxiety score was significantly higher compared to BL6 animals (STRAIN: *F*_1_,_69_ = 6.91, *p = 0.011). No difference between sexes was detected (SEX: *F*_1_,_69_ = 1.71, *p* = 0.196). **(B)** Unified sociability score. The 129 sociability score was significantly higher compared to BL6 (STRAIN: *F*_1_,_69_ = 10.90, **p = 0.002). No difference between sexes was detected (SEX: *F*_1_,_69_ = 0.30, *p* = 0.583). **(C)** Test score principal component cluster plot for PC1 and PC2. **(D)** Mean test score principle component 1 values by group (26.1% of variability). Clustering of strains was significant for PC1 (STRAIN: *F*_1_,_69_ = 28.45, ***p < 0.001). No difference between sexes was detected (SEX: *F*_1_,_69_ = 0.09, *p* = 0.766). Error bars represent ± SEM.

129 mice demonstrated a higher anxiety score compared to the BL6 mice, with no difference between males (STRAIN: *F*_1_,_69_ = 6.91, *p* = 0.011, Figure 3A). 129 mice also exhibited a greater level of social interaction compared to the BL6 mice (STRAIN: *F*_1_,_69_ = 10.90, *p* = 0.002, Figure 3B). No difference in the integrated anxiety or sociability scores between sexes was identified [SEX(Anxiety): *F*_1_,_69_ = 1.71, *p* = 0.196 and SEX(Sociability): *F*_1_,_69_ = 0.30, *p* = 0.583 respectively].

PCA of individual animal scores based on all behavioral tests conducted demonstrated significant clustering of mice in PC1 into strain groups, although separate clustering of sexes was not detected (STRAIN: *F*_1_,_69_ = 28.45, *p* < 0.001 and SEX: *F*_1_,_69_ = 0.09, *p* = 0.766 respectively, Figures 3C,D).

### Anxiety and Stress-Related Tests

**FIGURE 4:**
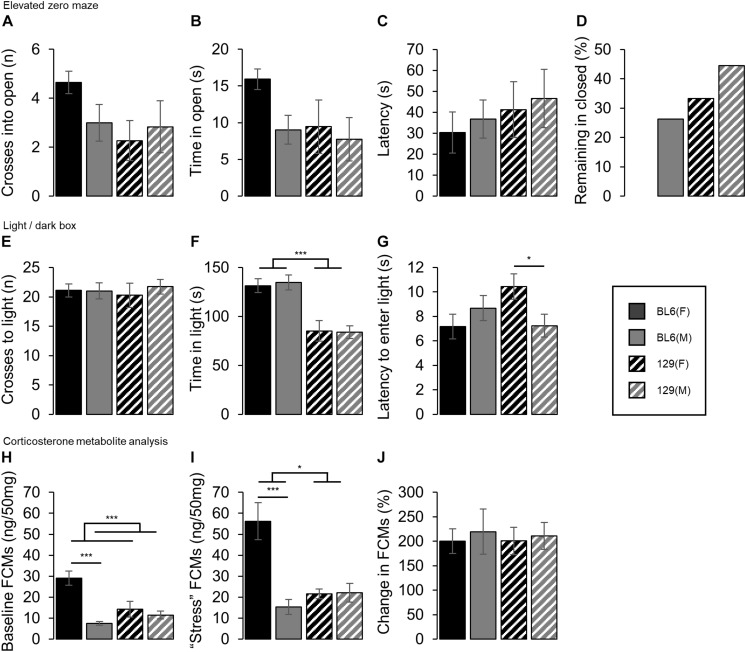
Individual anxiety and stress-related tests. **(A)** No strain or sex differences in the number of crosses into the open section of the EZM were detected. **(B)** Time spent in the open section of the elevated zero maze. No strain or sex effects were found. **(C)** Latency to first enter the open section of the elevated zero maze. There was no difference in the time to enter either between strains or between sexes. **(D)** Proportion of each group that did not enter the open section of the elevated zero maze. A greater proportion of the 129 females (33%) and males (56%) remained in the closed section of the maze for the entire duration of the test, compared to the BL6 females (0%) and males (30%) respectively. **(E)** Number of crosses into the light chamber of the light/dark box. No effect of strain or sex were detected in the number of crosses **(F)** Time spent in the light chamber of the light/dark box. BL6 mice spent longer in the light chamber than the 129s (STRAIN: *F*_1_,_69_ = 42.11, ***p < 0.001), however no sex differences were found. **(G)** Latency to first enter the light chamber of the light/dark box. Female 129s took longer to enter the light chamber compared to male 129s [STRAIN*SEX: *F*_1_,_69_ = 6.01, *p* = 0.017, 129(SEX): *t*_31_ = 2.30, *p = 0.014], but no difference was seen between the BL6 mice. **(H)** Baseline concentrations of fecal corticosterone metabolites (FCMs). Females excreted higher FCM levels than the males (SEX: *F*_1_,_44_ = 19.79, ***p < 0.001). Higher FCM concentrations were excreted by the female BL6 mice compared to the male BL6 mice [STRAIN*SEX: *F*_1_,_44_ = 11.85, *p* = 0.001 and BL6(SEX): *t*_12_ = 6.51, ***p < 0.001], but no difference in baseline levels were seen between the 129 mice. **(I)** FCM concentrations under stressed conditions. Females excreted higher FCM levels than the males (SEX: *F*_1_,_43_ = 13.72, ***p < 0.001). Higher FCM concentrations were excreted by theBL6 mice compared to the 129 mice (STRAIN: *F*_1_,_43_ = 6.54, *p* = 0.014), although this was driven by a higher FCM concentration in BL6 females compared to males which was absent between the 129s [STRAIN*SEX: *F*_1_,_43_ = 14.58, *p* < 0.001 and BL6(SEX): *t*_14_ = 4.29, ***p < 0.001]. **(J)** Percentage change in FCM concentrations under stressed conditions compared to baseline. No difference between sexes or strains was found. Error bars represent ± SEM.

Individual test results are shown in Figure 4.

No difference in the number of crosses into the open section of the EZM was detected between the BL6 and 129 animals (STRAIN: *F*_1_,_68_ = 2.92, *p* = 0.092, Figure 4A). No strain differences were detected for either females or males in the time spent in the open section (STRAIN: *F*_1_,_68_ = 2.75, *p* = 0.102, Figure 4B) or the latency to enter (STRAIN: and *F*_1_,_50_ = 1.25, *p* = 0.269, Figure 4C). However, a greater proportion of the 129 females (33%) and males (56%) remained in the closed section of the maze for the entire duration of the test, compared to the BL6 females (0%) and males (30%) respectively, Figure 4D. No main effects of sex were found for the number of crosses (SEX: *F*_1_,_68_ = 0.53, *p* = 0.469), time spent in the open section (SEX: *F*_1_,_68_ = 3.35, *p* = 0.072) or latency to enter open (SEX: *F*_1_,_50_ = 0.40, *p* = 0.530). A greater proportion of males (34%) remained in the closed section for the duration of the test than the females (14%).

No differences in the number of crosses into the light chamber of the light/dark box were detected between strains (STRAIN: *F*_1_,_69_ = 0.00, *p* = 0.968, Figure 4E). In addition, no effect of sex was observed (SEX: *F*_1_,_69_ = 0.25, *p* = 0.620).

BL6 mice spent longer in the light chamber than the 129s (STRAIN: *F*_1_,_69_ = 42.11, *p* < 0.001, Figure 4F). No sex differences were found (SEX: *F*_1_,_69_ = 0.02, *p* = 0.895). No main effects of strain or sex in the latency to enter the light chamber were found (STRAIN: *F*_1_,_69_ = 0.91, *p* = 0.343 and SEX: *F*_1_,_69_ = 0.77, *p* = 0.383, Figure 4G) however a STRAIN^∗^SEX interaction was detected, which was driven by female 129 mice taking significantly longer to enter the light chamber than the male 129s, whilst no difference was found between the BL6s [STRAIN^∗^SEX: *F*_1_,_69_ = 6.01, *p* = 0.017, 129(SEX): *t*_31_ = 2.30, *p* = 0.014 and BL6(SEX): *t*_38_ = 1.06, *p* = 0.851].

Analysis of FCMs revealed that female mice showed higher concentrations compared to males in both baseline and ‘stressed’ conditions [SEX(Baseline): *F*_1_,_44_ = 19.79, *p* < 0.001, Figure 4H, and SEX(Stressed): *F*_1_,_43_ = 13.72, *p* < 0.001, Figure 4I, respectively]. A STRAIN^∗^SEX interaction, but no main effect of strain was seen under baseline conditions (STRAIN^∗^SEX: *F*_1_,_44_ = 11.85, *p* = 0.001 and STRAIN: *F*_1_,_44_ = 3.92, *p* = 0.054 respectively), driven by a higher FCM concentration baseline in BL6 females compared to males which was absent between the 129s [BL6(SEX): *t*_12_ = 6.51, *p* < 0.001 and 129(SEX): *t*_16_ = 0.66, *p* = 0.260 respectively]. Under ‘stressed’ conditions both a main effect of strain and a STRAIN^∗^SEX interaction were observed (STRAIN: *F*_1_,_43_ = 6.54, *p* = 0.014 and STRAIN^∗^SEX: *F*_1_,_43_ = 14.58, *p* < 0.001 respectively), again driven by a higher FCM concentration in BL6 females compared to males which was absent between the 129s [BL6(SEX): *t*_14_ = 4.29, *p* < 0.001 and 129(SEX): *t*_17_ = 0.12, *p* = 0.549 respectively]. No differences in the change in FCM levels between baseline and stressed conditions were found between any groups (SEX(%Change): *F*_1_,_43_ = 0.20, *p* = 0.654, STRAIN: *F*_1_,_43_ = 0.02, *p* = 0.901, Figure 4J).

### Sociability-Related Tests

**FIGURE 5:**
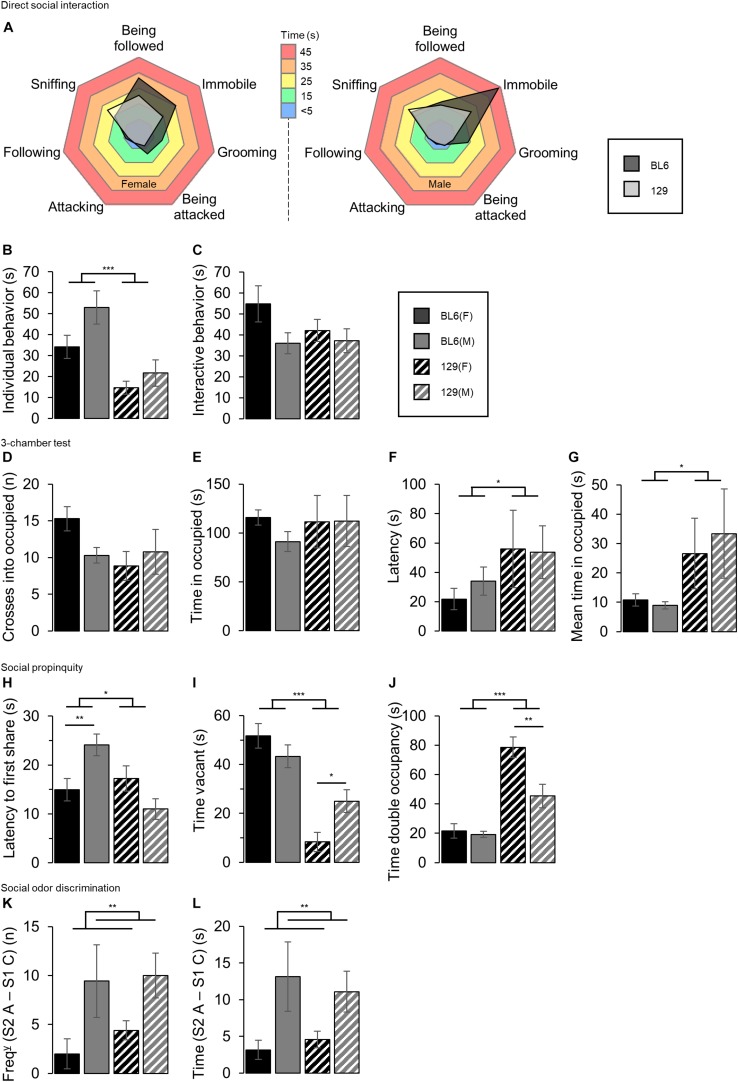
Individual sociability tests. **(A)** Ethogram charts of direct social interaction task behaviors. BL6 mice’s time allocation was more skewed toward grooming behavior, being immobile or being followed by the host compared to the 129 mice. **(B)** Time spent performing ‘individual’ behaviors. The male mice spent a greater amount of time on non-interactive ‘individual behaviors’ than the females (SEX: *F*_1_,_69_ = 4.70, *p* = 0.034) and BL6 mice spent a greater amount of time performing individual behaviors than the 129s (STRAIN: *F*_1_,_69_ = 18.16, ***p < 0.001). **(C)** Time spent performing ‘interactive’ behaviors. No difference between sexes, nor between strains in the time spent interacting in the direct social interaction task was shown. **(D)** Number of crosses into the occupied chamber of the 3-chamber test. No effect of sex or strain was detected. **(E)** Total time spent in the occupied chamber of the 3-chamber test. No differences between sexes or strain were detected. **(F)** Latency to enter the occupied chamber of the 3-chamber test. 129 mice took longer to enter the occupied chamber compared to the BL6 mice (STRAIN: *F*_1_,_62_ = 4.24, *p = 0.044) No differences between sexes were detected. **(G)** Mean time spent per visit to occupied chamber in the 3-chamber test. The 129 mice stayed longer in the occupied chamber per visit than the BL6s, although no sex differences were observed (STRAIN: *F*_1_,_69_ = 5.43, *p = 0.023). **(H)** Latency to first share the tube in the social propinquity test. BL6 mice took longer to share the tube than the 129s (STRAIN: *F*_1_,_60_ = 6.54, *p = 0.0.013). Compared to the BL6 males, the BL6 females were quicker to share the tube [STRAIN*SEX: *F*_1_,_60_ = 13.36, *p* < 0.001, BL6(SEX): *t*_38_ = 2.86, **p = 0.007], however this was not seen in the 129s. **(I)** Duration for which the tube was vacant in the social propinquity task. The time vacant was significantly greater for the BL6 mice compared to the 129s (STRAIN: *F*_1_,_68_ = 47.43, ***p < 0.001). The tube was vacant for a greater amount of time with the male 129s compared to the female 129s [STRAIN*SEX: *F*_1_,_68_ = 7.79, *p* = 0.007, 129(SEX): *t*_30_ = 2.64, *p = 0.013], although no differences were seen between the BL6 mice. **(J)** Time spent sharing the tube in the social propinquity test. The total time of double occupancy was increased in the 129 mice compared to the BL6s (STRAIN: *F*_1_,_68_ = 59.84, ***p < 0.001), and the female mice spent more time sharing the tube than the males (SEX: *F*_1_,_68_ = 11.04, *p* = 0.001). The 129 females shared for longer compared to the male 129s [STRAIN*SEX: *F*_1_,_68_ = 8.16, *p* = 0.006, 129(SEX): *t*_30_ = 3.03, **p = 0.005], but no differences between the BL6 mice were seen. **(K)** Difference in the number of visits between a novel social odor [Social odor 2, presentation A (S2 A)] and a familiar social odor [Social odor 1, presentation C (S1 C)]. The male mice increased the frequency of visits to the odor when a ‘familiar’ social odor was replaced by a novel one to a greater extent than the female [SEX(FREQ): *F*_1_,_60_ = 9.42, **p = 0.003]. No difference was detected between strains. **(L)** Difference in the time spent exploring a novel social odor [Social odor 2, presentation A (S2 A)] and a familiar social odor [Social odor 1, presentation C (S1 C)]. Male mice increased the time spent exploring the odor to a greater extent than the females [SEX(TIME): *F*_1_,_60_ = 10.16, **p = 0.002]. No difference was detected between strains. Error bars represent ± SEM.

Individual sociability-related test results are shown in Figure 5.

The ethogram charts of direct social interaction task behaviors demonstrate a tendency for the BL6 mice to spend a greater amount of time grooming, immobile or being followed by the host compared to the 129 mice, Figure 5A. The male mice spent a greater amount of time on non-interactive ‘individual behaviors’ than the females (SEX: *F*_1_,_69_ = 4.70, *p* = 0.034, Figure 5B), and BL6 mice spent a greater amount of time performing individual behaviors than the 129s (STRAIN: *F*_1_,_69_ = 18.16, *p* < 0.001). No difference between sexes, nor between strains in the time spent interacting in the direct social interaction task was shown (SEX: *F*_1_,_69_ = 3.60, *p* < 0.062, and STRAIN: *F*_1_,_69_ = 0.85, *p* = 0.359).

The 3-chamber test did not show a main effect of sex in either the number of crosses into, the time spent in, latency to enter or the mean time per visit to the occupied chamber (SEX: *F*_1_,_69_ = 0.64, *p* = 0.427, Figure 5D, SEX: *F*_1_,_69_ = 0.46, *p* = 0.498, Figure 5E, SEX: *F*_1_,_62_ = 0.15, *p* = 0.703, Figure 5F, and SEX: *F*_1_,_69_ = 0.08, *p* = 0.773, Figure 5G, respectively). Strain differences were not detected in either the time to cross into or time spent in the occupied chamber, however, the BL6 mice were quicker to enter compared to the 129s (STRAIN: *F*_1_,_69_ = 2.36, *p* = 0.129, STRAIN: *F*_1_,_69_ = 0.23, *p* = 0.635 and STRAIN: *F*_1_,_62_ = 4.24, *p* = 0.044 respectively). The 129 mice stayed longer in the occupied chamber per visit than the BL6s, although no sex differences were observed (STRAIN: *F*_1_,_69_ = 5.43, *p* = 0.023 and SEX: *F*_1_,_69_ = 0.08, *p* = 0.773 respectively).

The time taken to first share the tube was greater for the BL6 mice than the 129s in the social propinquity task (STRAIN: *F*_1_,_60_ = 6.54, *p* = 0.0.013, Figure 5H), and whilst no main effect of sex was detected, a STAIN^∗^SEX interaction was significant (SEX: *F*_1_,_60_ = 0.49, *p* = 0.487 and STRAIN^∗^SEX: *F*_1_,_60_ = 13.36, *p* < 0.001 respectively), driven by the male BL6 mice taking longer to first share than the female BL6s [BL6(SEX): *t*_38_ = 2.86, *p* = 0.007]. No difference in between the 129 groups was found [BL6(SEX): *t*_22_ = 1.88, *p* = 0.073].

The total time for which the tube was vacant was significantly greater for the BL6 compared to the 129 mice (STRAIN: *F*_1_,_68_ = 47.43, *p* < 0.001, Figure 5I), and whilst there was no main effect of sex, a STRAIN^∗^SEX interaction was detected SEX: *F*_1_,_68_ = 0.86, *p* = 0.356 and STRAIN^∗^SEX: *F*_1_,_68_ = 7.79, *p* = 0.007 respectively). *Post hoc* testing showed that the tube was vacant for less time for the female 129s compared to the 129 males, and no difference between the BL6 groups [129(SEX): *t*_30_ = 2.64, *p* = 0.013, BL6(SEX): *t*_38_ = 1.22, *p* = 0.231].

The total time of double occupancy was increased in the 129 mice compared to the BL6s (STRAIN: *F*_1_,_68_ = 59.84, *p* < 0.001, Figure 5J). Compared to the males, the females spent longer sharing the tube (SEX: *F*_1_,_68_ = 11.04, *p* < 0.001), although this difference appeared to be driven by the propensity of the 129 females to share for longer compared to the males since there was no sex difference detected in the BL6s [STRAIN^∗^SEX: *F*_1_,_68_ = 8.16, *p* = 0.006, 129(SEX): *t*_30_ = 3.03, *p* = 0.005 and BL6(SEX): *t*_26_ = 0.47, *p* = 0.645].

During the odor discrimination task, the male mice increased the frequency of visits to the odor, and the time spent exploring the odor when a ‘familiar’ social odor was replaced by a novel one to a greater extent than the female [SEX(FREQ): *F*_1_,_60_ = 9.42, *p* = 0.003, Figure 5K and SEX(TIME): *F*_1_,_60_ = 10.16, *p* = 0.002, Figure 5L], however, no difference was detected between strains for either the increase in odor visit frequency (STRAIN: *F*_1_,_60_ = 0.49, *p* = 0.488) or odor visit time (STRAIN: *F*_1_,_60_ = 0.01, *p* = 0.905).

## Discussion

Behavioral traits in animal models are often subtle and complex, and detecting replicable disturbances can be challenging. The multifaceted nature of any behavior necessitates the use of multiple tests to obtain a truly reflective assessment (Belzung and Griebel, 2001). Anxiety-related behaviors, for example, are known to arise from several different neuronal systems (Ferreri et al., 2011), may present in slightly different ways or intensities (Montkowski et al., 1997) and certain behavioral tests may not be sensitive enough to detect subtle changes. The data presented here, from three different tests of anxiety and stress (the elevated zero maze, light/dark box and FCM analysis), show how disparate outcomes can be generated depending on the test used. Many studies will often probe behaviors using just one test. However, as we show here, one test may not be adequate to detect true effects. A major problem with conducting multiple tests is that the probability of generating type I errors increases, and while this can be corrected for by using more rigorous statistical thresholds, it is not ideal (Shaffer, 1995). The current study presents a novel method for utilizing a broad range of behavioral probes and consolidating all outcome measures to provide a simple and comparable score for each type of behavioral trait being investigated. The combined score incorporates equal input from each test performed whilst minimizing the probability of reporting chance results as significant effects or missing subtle behavioral disturbances.

Principal component analysis of the test outcome measures was able to effectively categorize their contribution to the traits being probed, thus justifying the positive/negative allocation of behaviors to each test score. The resulting unified scores were able to detect a difference in the degree of anxiety-related behavior that female 129s exhibit compared to female BL6 mice, a result which was reflected significantly in some but not all behavioral tests or outcome measures. This is congruent with the conclusion of several previous strain comparison studies, albeit with different tests or test combinations (Rogers et al., 1999; Võikar et al., 2001; Abramov et al., 2008; Harms et al., 2008). Interestingly, contrary to these previous reports, the unified score for anxiety did not detect a difference between the male mice. In fact, it was only the light/dark box test ‘time in light’ measure that demonstrated a significant increase in anxiety-like behavior in the male 129s – a test that is frequently used as the sole measure for reporting this behavior. The data suggests that anxiety-related behaviors in the males may be affected by a more restricted range of aspects of anxiety, whereas the female mice may present a more generalized anxious state. This result may reflect the dimorphic manifestation of anxiety-related symptoms in people, where women are twice as likely to be diagnosed with anxiety related conditions than men (Martín-Merino et al., 2009). Female mice demonstrated a higher baseline concentration of corticosterone metabolites in their feces compared to the males. Whilst high levels of this stress hormone may suggest female mice are generally more stressed than the males, it was observed that following exposure to predator odor all groups showed a similar proportional increase in FCMs. Other than ‘amount’ of stress *per se*, sex differences in the FCM baseline measures could be explained by differences in metabolism or excretion (Touma et al., 2003, 2004), or due to disparities in the proportion of metabolite types being produced, since only one marker was used in the current study.

The unified scores for sociability detected a greater tendency for interaction in both sexes of the 129s compared to the BL6 mice, a result which was less clearly discerned from the individual tests. This trait is consistent with direct social interaction findings presented by Hughes (1989) and Harms et al. (2008) who observed that 129/Sv mice spent more time engaged in social interaction than other strains. Importantly, PCA of the individual test scores revealed significant clustering in PC1 of the experimental groups, indicating that the effect of ‘group’ may be the primary factor affecting the greatest variance within the data set and thus supporting the effectiveness of the normalized unified scoring system.

The data presented demonstrate a proof of concept for the use of unified scores to present a clear behavioral phenotype using data from multiple tests – the results of which are validated by a broad range of previously published studies probing aspects of these behaviors in these strains (Hughes, 1989; Crawley et al., 1997; Montkowski et al., 1997; Belzung and Griebel, 2001; Liu and Gershenfeld, 2001; Võikar et al., 2001; Touma et al., 2004; Sankoorikal et al., 2006; Abramov et al., 2008; Yang et al., 2011; Lorenz et al., 2013), however, further validation of the sensitivity of the unified scoring method through pharmacological studies would be beneficial. These studies could also be used to determine the optimum number of different tests to include within a test battery for each particular behavioral trait. The current study used at least 3 different tests to probe each behavioral trait, which resulted in a clear result backed up by previous studies, however, it would be useful to investigate when the benefits of including more tests to increase the integrity of the data is outweighed by the amount of work and logistical practicalities involved.

The underlying method presented here for anxiety-related and social-related tests has potential to be applied to other aspects of behavior by amalgamating related output measures probing behavior outcomes such as depression, cognition, or motor deficiencies. For example, combining measures of the tail suspension test, forced swim test and hedonistic response could provide a more accurate indication of the depressive-like state of an individual than either one on its own, and could therefore help to improve the reporting of behavioral results and subsequently reproducibility, for which there is growing call (Landis et al., 2012). Furthermore, since all data is normalized to a score between 0 and 1 based on comparisons within a dataset, it allows the unified scores to be compared between experiments and labs by reducing the influence of environmental/temporal variations on basic outcome measures. Importantly, the lack of sex effects between the female and male scores demonstrate a counterargument to the common presumption that female mice are less suited to behavioral testing. The normalization of data into unified comparative scores could further support the use of both sexes for investigation of sexual dimorphic patterns of behavior.

## Conclusion

The unified behavioral score can incorporate a broad range of behavioral probes and present a simple comparable score reflective of generalized behavioral traits. The method is sensitive enough to detect subtle differences in complex behaviors that may be easily missed by individual tests. This novel approach aimed at presenting and comparing behavioral traits will be a useful tool for exploring the effects of genetic manipulations, disease, adversity or interventions in animal models of psychiatric disorders.

## Data Availability Statement

The raw, transformed and unified score datasets for this study can be found in the FigShare Repository,

## Ethics Statement

The animal study was reviewed and approved by the local Cardiff University Ethics Review Committee.

## Author Contributions

DH, RJ, and AI contributed to the concept and design of the study. DH, HC, HT, and RB-S conducted the behavioral tests. RP and CT corticosterone metabolite analyses. DH wrote the draft manuscript. All authors contributed to the manuscript revision, read and approved the submitted version. RJ acquired the funding.

## Conflict of Interest

The authors declare that the research was conducted in the absence of any commercial or financial relationships that could be construed as a potential conflict of interest.
